# Promoting hEalthy Diet and Active Lifestyle (PEDAL): a protocol for the development and feasibility study of a multicomponent intervention among primary school children in Singapore

**DOI:** 10.1186/s40814-024-01479-3

**Published:** 2024-03-23

**Authors:** Cindy Mei Jun Chan, Falk Müller-Riemenschneider, Michael Yong Hwa Chia, Zoe Jane-Lara Hildon, Mary Foong-Fong Chong

**Affiliations:** 1 Saw Swee Hock School of Public Health, National University of Singapore and National University Health System, Tahir Foundation Building (MD1), 12 Science Drive 2, #09-01Q, Singapore, 117549 Singapore; 2 Center for Digital Health, Berlin Institute of Health (BIH), Charité-Universitatsmedizin Berlin, Berlin, Germany; 3 Physical Education & Sports Science Academic Group, National Institute of Education, Nanyang Technological University, Singapore, Singapore; 4 Singapore Institute for Clinical Sciences, Agency for Science, Technology and Research, Singapore, Singapore

**Keywords:** Healthy eating, Sedentary behaviour, School-based intervention, Feasibility, Primary school, Multicomponent

## Abstract

**Background:**

Setting healthy lifestyle habits during the formative years of childhood is critical as habits can track to adulthood and help prevent obesity and chronic disease risks in later life. While multicomponent interventions have been shown to be effective in changing the lifestyle behaviours of children, there is a limited understanding of the feasibility of such interventions in primary schools in Singapore. A multiphase mixed method study was conducted to develop and examine the feasibility of a theory-based multicomponent school-based intervention—Promoting hEatlthy Eating and Active Lifestyle (PEDAL).

**Methods:**

Underpinned by Kincaid’s ideation model, the PEDAL intervention was developed to increase fruit and vegetable consumption and decrease sedentary behaviours among children. This study consists of three phases. Phase 1 details the development of PEDAL, which consists of four components: (A) a series of interactive health education lessons, (B) actionable home activities to support habit formation, (C) parental/guardian engagement, and (D) optimising the school environment. In Phase 2, components A and B of PEDAL were implemented in two public, co-educational primary schools among Primary 5 students (aged 10–12 years) in Singapore. Data was collected quantitatively using questionnaires and qualitatively using focus group discussions (FGDs) with students and teachers. The feasibility dimensions of components A and B, including recruitment capability, data collection, social validity, and practicality were examined, and ideation on healthy eating and physical activity was explored. In Phase 3, the full PEDAL intervention was pilot-tested in two other public, co-education primary schools with the same target population, using a concurrent mixed method quasi-experimental study design. Feasibility dimensions and potential effectiveness of the intervention will be assessed.

**Discussion:**

This study will provide insights into the feasibility of PEDAL and inform its refinement. Findings from the pilot test will guide the planning of a larger-scale definitive trial.

**Trial registration:**

Registered with ISRCTN registry (ISRCTN16114046) on 16 October 2022.

**Supplementary Information:**

The online version contains supplementary material available at 10.1186/s40814-024-01479-3.

## Background

Adolescence, defined by WHO as ages between 10 and 19 years, is one of the most rapid, dynamic, and formative phases of human development [1]. It provides a window of opportunity for establishing healthy lifestyle habits that can track to adulthood [2, 3]. Yet, increasing evidence indicates that, globally, older children and adolescents do not meet dietary and movement behaviour (comprising physical activity, sedentary behaviour, and sleep) guidelines, thus contributing to the obesity epidemic [3, 4]. This is of concern as childhood obesity strongly predicts adult obesity and is associated with health problems such as diabetes and cardiovascular diseases [5–7]. Obese children also tend to have lower levels of self-esteem, a higher prevalence of depressive symptoms, and be socially marginalised with conditions persisting into adulthood [8, 9]. Recent evidence indicated an accelerated trend in children’s and adolescents’ BMI in parts of Asia [10]. The prevalence of overweight among children and adolescents (age 5–19 years) in Singapore stands at 22% in 2021, ranking third highest in the ASEAN region [11].

In Singapore, a significant proportion of primary school children were found not meeting healthy behaviour guidelines [12]. Many had diets that were lacking in fruits, vegetables, and whole grains while high in sodium and added sugar [12, 13]. The recent national Report Card on physical activity level among children and adolescents reported that only 44% and 41% of children and adolescents met the moderate-to-vigorous physical activity (MVPA) guidelines of at least 60 min daily and the recreational screen time guidelines of ≤120 min, respectively [14]. To improve children’s eating and movement behaviours, schools have been a popular means due to their potential for mass reach [15] and cost-effectiveness [16]. Recent evidence suggests that school-based interventions should be multicomponent to be effective in changing lifestyle behaviours. This means targeting more than one lifestyle behaviour simultaneously, plus engaging the family and providing environmental support [17–19]. This aligns with empirical evidence that lifestyle behaviours tend to cluster (e.g. the clustering of poor dietary habits and low levels of physical activity), indicating that a single behaviour change does not necessarily result in an overall healthy lifestyle [20].

**Fig. 1:**
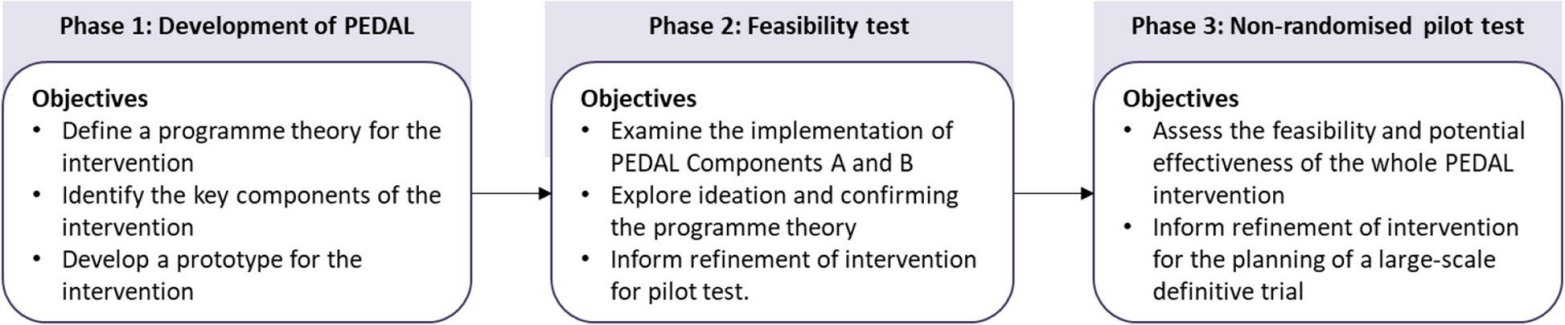
Description of the multiphase mixed method study to develop and test the PEDAL intervention

While school-based lifestyle interventions with a multicomponent design have been conducted in Asia and Singapore, there is a paucity of published studies on the effectiveness of these interventions on obesity outcome measures (e.g. body mass index, waist circumference) [21–23]. Findings were also reported to be mixed [21, 22]. While some studies found substantial effects on health knowledge and lifestyle behaviours, behavioural outcomes were not often reported [21, 22]. Furthermore, as intervention effectiveness has shown to vary by context, information on the real-world adoption, implementation, and sustainability of multicomponent school-based interventions is lacking in the Asian context [21, 24]. Using a theory-based intervention and testing the programme theory will help understand probable “active ingredients” that make the intervention effective [1, 19]. Thus, a multiphase mixed method study was conducted to develop and examine the feasibility of a theory-based multicomponent school-based intervention—Promoting hEalthy Eating and Active Lifestyle (PEDAL) in Singapore. This study consists of three phases: a development phase (Phase 1) and two feasibility phases (Phases 2 and 3) as detailed in Fig. 1. Briefly, Phase 1 focused on the development of the intervention content, materials, and activities, while Phase 2 examined the feasibility of implementing the interactive health education lessons (Component A) and home activities (Component B) and explored ideation on healthy eating and physical activity. In Phase 3, a pilot study was conducted to examine the feasibility and potential effectiveness of the whole PEDAL intervention. This paper is reported according to the Standard Protocol Items: Recommendations for Interventional Trials (SPIRIT) guidelines [25] (Additional File 1). Findings from this study will provide critical information on the refinement of the intervention before the conduct of a definitive trial to determine the effectiveness of the intervention.

## Methods

### Phase 1: Development of PEDAL

The specific aims of PEDAL are to increase fruit and vegetable intake, increase time spent on MVPA, and decrease sedentary time spent. PEDAL is underpinned by Kincaid’s ideation model, a metatheory of health communication that posits that successful interaction between resources, environment, and ideation factors would lead to behaviour change [26]. In this model, ideational factors are grouped into three domains: cognitive, emotional, and social determinants of behaviour, and the likelihood of practising a recommended behaviour increases when more factors from the three domains apply to an individual [27, 28].

**Fig. 2:**
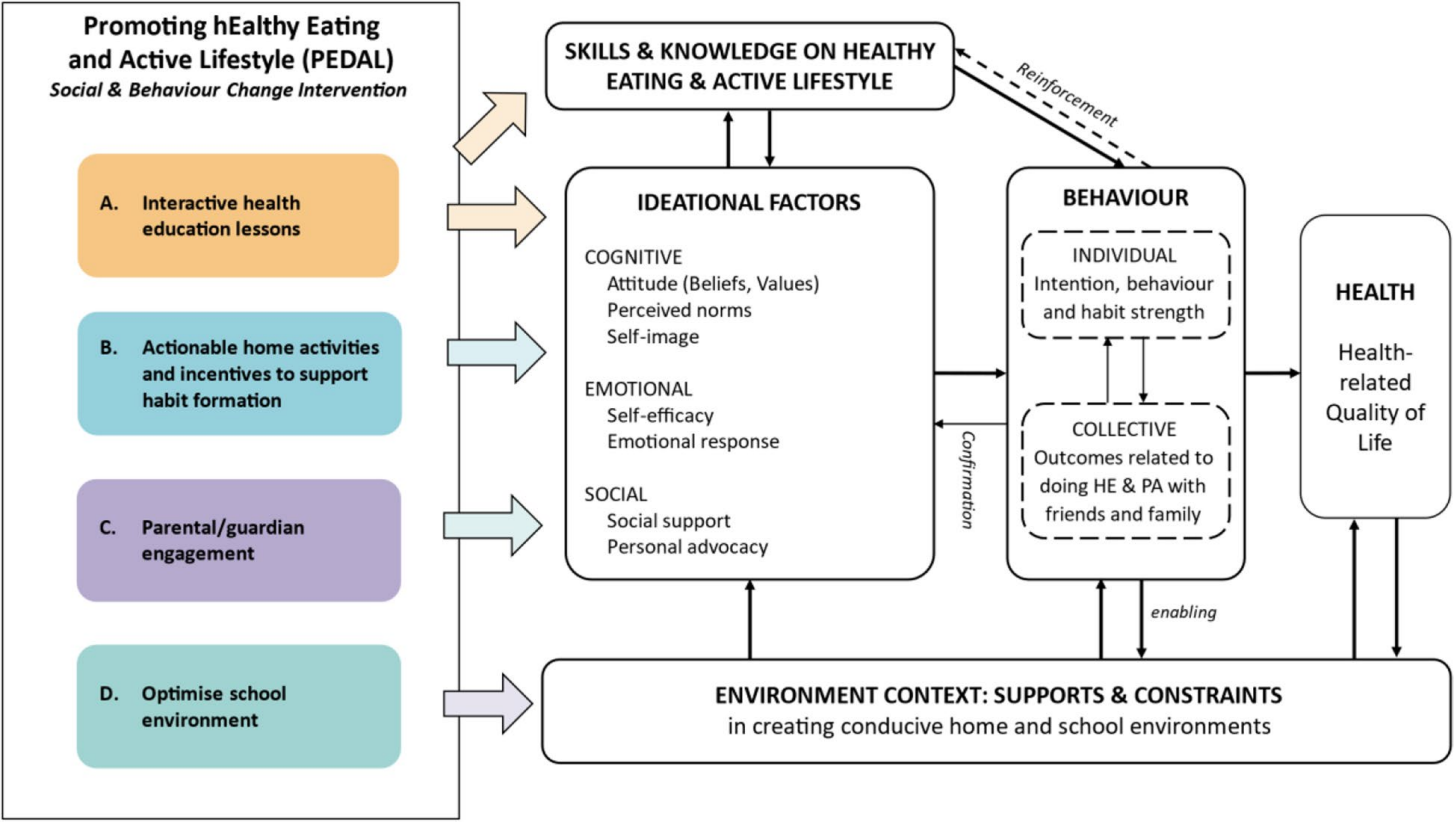
Illustration of the programme theory for the PEDAL intervention

Based on current literature and previous work by the team [19, 20, 29–31], factors that can potentially influence children’s healthy eating and activity behaviours include knowledge, home and school environment, attitudes, self-efficacy, emotional response, social norms and support, behavioural intentions, and habit strength. With this knowledge, a programme theory was developed for the intervention (Fig. 2), with four key components targeting these factors. The four components include a series of interactive health education lessons (Component A), actionable home activities to support habit formation (Component B), involving parents or guardians (Component C), and optimising the school environment to support and reinforce healthy eating and lifestyle behaviours (Component D). Details of these components are described below and summarised in Table 1.

**Table 1:** Summary of intervention components and behaviour change techniques and constructs applied

Intervention component	Content	Behaviour change techniques (coded according to the BCTTv1 [32])	Ideational variables (constructs)
A. Interactive health education lessons	• Introduction of physically active and interactive health education lessons • Providing personalised lifestyle reports • Introducing goal setting and habit formation into the lessons	1.1 Goal setting (behaviour); 1.2 problem solving (coping planning); 1.4 action planning; 1.9 commitment; 2.2 feedback on behaviour; 4.1 instruction on how to perform a behaviour; 5.1 information about health consequences; 5.6 information about emotional consequences; 9.1 credible source	• Knowledge/skills • Attitudes • Self-efficacy • Emotional response
B. Actionable home activities and incentives to support habit formation	• Complete a variety of activities that can be repeated to support habit formation • Incentivising task completion	3.1 Social support (practical); 8.1 behavioural practice/ rehearsal; 8.3 habit formation; 10.1 material incentive (behaviour); 10.2 material reward (behaviour)	• Knowledge/skills • Attitudes • Self-efficacy
C. Parental/guardian engagement	• Engaging parents/guardians in home activities • Sharing of children’s personalised lifestyle reports with parents/guardians • Sharing of lesson materials with parents/guardians	2.2 Feedback on behaviour; 3.2 social support (practical)	• Attitudes • Social support
D. Optimise school environment	• Level-wide activities during recess to increase awareness of fruits and vegetables and ideas to break up sitting time • Working with canteen vendors on the providence and placements of cues to encourage fruit and vegetable intake.	7.1 Prompt/cues; 6.2 social comparison; 10.4 social reward	• Attitudes • Perceived norms • Emotional response • Environment support

An advisory committee comprising experienced health and education professionals from the Ministry of Education (MOE), the Health Promotion Board (HPB), the National Institute of Education, and the Saw Swee Hock School of Public Health was formed to provide guidance and input towards the development of PEDAL.

#### Component A: Interactive health education lessons

Consisting of five interactive face-to-face lessons, the PEDAL health education lessons were developed to cover topics on healthy eating, reducing sedentary behaviours, and habit formation (see link for introductory video: https://bit.ly/pedal-intro, and Table 2 for a summary of lesson topic and content). The concept of habit formation was included as increasing evidence has demonstrated that habit is a more important predictor of a behaviour (e.g. fruit and vegetable consumption) than intentions [33, 34] and the need to identify contingency plans that help support existing habits [35]. Adapting the concept from a habit-based healthy feeding intervention [36], students were taught to incorporate strategies such as goal setting, self-monitoring (through understanding triggers and circumstances), and self-efficacy in the PEDAL lessons. To help facilitate students’ goal setting, which provides a starting point for making specific behavioural changes, students received feedback on their lifestyle behaviours based on their baseline lifestyle behaviour data collected. To reduce sedentary time, health education lessons, typically conducted with seated students, were modified to incorporate standing and movement activities during lessons to break up sitting time [37]. This approach has the triple benefits of reducing students’ sitting time, teaching them the concept of reducing sedentary behaviour as well as being more interactive, to better engage the students and change their perception of health education lessons.

**Table 2:** Topics covered in the health education lessons

Lesson	Topic	Content and activities
1	Calorie balance and nutrient density	• Educational video on energy density and portion sizes; • Interactive and active stand-up game to reinforce concepts learnt and bust diet myths Video links: https://bit.ly/pedal-caloriebalance, https://bit.ly/pedal-nutrientdensity (See Additional File 2 for snapshots of the lesson videos)
2	Habit formation	• Educational video on the habit formation concept; • Worksheets on goal setting based on class and individual feedback on diet and lifestyle; • Worksheets on setting action plans for diet and movement. Video link: https://bit.ly/pedal-habitformation (See Additional File 2 for snapshots of the lesson video)
3	Home assignment—PEDAL card	• Introduction to PEDAL card home activities to encourage repetition of healthy behaviours and the point system for completing home activities. (See Additional File 3 for snapshots of the home assignment tasks)
4	Sit less, move more	• Educational video on sedentary behaviours and the impacts on the body, and tips on how to break up long hours of sitting; • Interactive and active stand-up game to reinforce concepts learnt Video link: https://bit.ly/pedal-sitlessmovemore (See Additional File 2 for snapshots of the lesson video)
5	Coping Plans	• Educational slides to introduce coping plans • Interactive and active stand-up game to identify coping plans to overcome common barriers faced when increasing fruit and vegetable intakes and reducing sedentary behaviours; (See Additional File 2 for snapshots of the lesson slides)

#### Component B: Actionable home activities and incentives to support habit formation

Children’s automaticity or habit in behaviour often arises from the repetition of behaviours in a stable context [38]. To encourage sustained behavioural change and form habits, students were given home activities to complete. These consisted of individual-led tasks or tasks to be completed with their friends, family members, or guardians at their own pace. They aimed to develop students’ skills and self-efficacy in achieving set behavioural goals by targeting cognitive domains of knowledge and understanding and skill domains such as functional, interactive, and critical skills [39]. Examples of tasks include identifying three nutrient-dense foods, learning from parents how to prepare fruits independently, engaging in physical activity with their family, and executing the diet and movement habit plans students set for themselves. The home activities, presented on a card (PEDAL card, see Additional File 3 for snapshot), were introduced to students during the health education lessons, and students were given at least 6 weeks to complete the activities.

Students were encouraged to complete and repeat these activities through the collection of points. They were awarded a badge of honour (Bronze, Silver, Gold) by the end of the intervention through the accumulation of a certain number of points. Previous fruit and vegetable intervention studies have demonstrated that using small incentives is an effective way to encourage children to eat fruits and vegetables, and these induced changes persisted (at least 2 months after), even when incentives are no longer offered [40].

### Component C: Parental/guardian engagement

According to current evidence, school-based interventions directly engaging parents are more effective than indirect strategies such as intervention-related newsletters [41]. Schools were encouraged to share feedback on students’ lifestyle behaviours with parents for this component. After completing all five teaching lessons, teachers shared the lesson video links with parents through the parent communication application. This serves to inform parents of their children’s lifestyle behavioural statuses and the health education concepts taught during the lessons and garner their support for their children’s participation in the intervention. As previously mentioned, a proportion of home activities were designed to encourage parental/family involvement so that children make behavioural changes with the support of at least one family member. Additionally, children were required to seek acknowledgement from their parents upon completing various activities, allowing parents to be aware of their children’s tasks and provide the necessary support.

#### Component D: Optimise school environment

There is strong evidence to demonstrate that intervening through education and environmental changes on both dietary and movement behaviours would enable sustainable and long-term behaviour change [42]. Hence, we planned to enhance children’s affective attitudes towards fruits and vegetables and reduce sedentary time by gamifying healthy eating and experiential learning through school-level-wide activities [34, 43]. With the help of the school’s Parent Support Group (PSG) and in collaboration with the Singapore Health Promotion Board, mini-games with different themes (e.g. guessing the fruit and vegetables through touch and smell, activity ideas/competition for breaking up sitting time, and a healthy lifestyle quiz) were conducted over at least two 30-min recess breaks during the intervention period. Students were awarded small tokens for completing the games.

Concomitantly, we worked with canteen vendors to develop practical and economical solutions to optimise the school food environment and reinforce the value of the current Healthy School Meals Programme (HSMP) [44]. For example, using choice architecture, such as the placement of foods and posters as cues, to remind students to ask for vegetables and take a portion of fruit. Needs assessments, through interviews, were conducted before the intervention commenced in both schools to help decide on practical solutions to optimise the canteen environment.

### Phase 2: Feasibility of PEDAL components A and B

#### Objectives

The objectives of this phase were to examine the feasibility of implementing components A and B of PEDAL and explore the experiences of ideation to confirm the intervention’s programme theory. Both qualitative and quantitative methods were used for this study. The findings from this phase informed the refinement of these two components and the evaluation tools used in Phase 3.

#### Key measures

##### Feasibility

Data were collected to assess five feasibility dimensions delineated by Gadke et al. [45]: recruitment capability, data collection procedures, social validity, practicality, and implementation. The recruitment capability dimension assesses the capability of successfully recruiting study participants in our study. Data collection procedures examine the appropriateness of data collection procedures and tools. Social validity assesses participants’ acceptability and satisfaction with the intervention. Practicality examines whether it is practical to implement the intervention with the available resources, time, training, and materials. Lastly, the implementation dimension examines the implementation fidelity. Progression criteria for each feasibility dimension were also pre-determined, guiding the decision and actions from Phases 2 to 3. Details of the feasibility dimensions and the progression criteria are shown in Table 3.

**Table 3:** Feasibility dimensions, questions, measures and progression criteria for Phases 2 and 3^a^

Feasibility dimension	Research questions	Measure(s)	Progression criteria
Recruitment capability	Can participants who will benefit from and who will implement intervention be identified?	• Recruitment and participation rates of students and teachers • Recruitment rates of canteen vendors^a^	• ≥ 50% consent rates • ≥ 50% of consented participants attended the lessons • ≥ 50% consent rates from canteen vendors^a^
Data collection procedures	Are data collection procedures and outcome measures appropriate and sensitive to change?	• Survey completion rates among students and teachers • Descriptive analysis of scores from outcome measures • Survey completion rates among parents	• ≥ 50% of consented participants completed the survey • Survey questions and scales are appropriate without a ceiling effect • ≥ 50% of consented parents completed the survey
Social validity	Do participants perceive the intervention as appropriate, reasonable, fair, and potentially effective?	• Post-intervention feedback surveys from teachers and students • Post-intervention FGDs with students and teachers • Post-intervention interviews with canteen vendors^a^	• ≥ 75% satisfaction from student’s and teacher’s feedback surveys • Positive experiences from students, teachers, and canteen vendors^a^ • Positive experiences from canteen vendors^a^
Practicality	Can the intervention be implemented with available resources, time, training, and materials?	• Post-intervention FGDs with students and teachers • Post-intervention interviews with canteen vendors^a^ • Number of parent volunteers participated in canteen activities^a^	• Good feedback on the resources provided • Low level of perceived barriers on implementation from teachers and canteen vendors^a^ • At least 5 parent volunteers participated in the canteen activities^a^
Implementation	Are teachers able to implement the intervention with fidelity (as intended by the research team)? Are the canteen posters still in place by the end of the intervention?^a^	• Teacher-reported implementation checklist • Class observations^a^ • Canteen environmental scans^a^	• ≥ 75% fidelity among teachers, based on the checklist • ≥ 75% fidelity among teachers, based on class observations^a^ • ≥ 75% of the posters are still in place at the end of the intervention^a^
Potential Effectiveness of the whole PEDAL intervention^a^	Is there preliminary evidence of potential for bringing about positive change? What influenced the change (or no change) among the students?^a^	• Student outcome data^a^ • Canteen environmental scans ^a^ • Post-intervention interviews with students and teachers^a^ • Post-intervention interviews with canteen vendors^a^	• Improvements in fruit and vegetable intakes^a^ • Improvements in MVPA min/day^a^ • Reduction in screen time min/day^a^ • Improvements in ideation factors^a^ • Improvements in student’s health-related quality of life^a^ • Perceived improvements in behaviour and increased motivation for behaviour change^a^

^a^Additional items measured in Phase 3

##### Exploring ideation on healthy eating and physical activity

To confirm the feasibility of the programme theory, ideation on healthy eating and physical activity was explored using qualitative methods. Focus group discussions (FGDs) were conducted at the end of the intervention with students who participated and teachers who delivered the lessons. During the FGD, ideation factors that drive or impede healthy eating and physical activity among children and how components A and B influenced these ideation factors were explored. The findings from this will also inform the appropriateness of measuring the ideation variables in the questionnaire.

#### Setting and participants

Upon completion of the intervention prototype, two co-educational public schools in Singapore were recruited by convenience sampling to implement components A and B (health education lessons and home activities) of PEDAL. Co-educational public schools were selected as these were the most common type of primary schools in Singapore. The participants were students from the Primary 5 level, typically aged between 10 and 11 years old, from each school. This age group was chosen based on previous research that found that children aged 10 and above were capable of independently recalling and reporting their past activities [13, 46]. Two schools were selected for the pilot test as the required sample size to establish feasibility was calculated to be 385. This was calculated using a confidence interval (CI) approach [47], with a 95% CI, a margin of error of 0.05, and an expected completion rate of 50% based on our previous experience conducting research studies in primary school. Given that a primary school typically consists of six to eight classes of 40 students per level, the recruitment of two schools will meet the required sample size calculated. As the intervention posed minimal risk and was conducted as part of the school's Physical and Health Education curriculum, all Primary 5 students in the two selected schools were eligible to participate.

Both components, A and B, were delivered by each school’s Primary 5 Physical Education (PE) teachers. Training and lesson materials (e.g. slides, videos, home activity instructions, activity booklets for students, and teacher’s guide) were provided by the research team to the PE teachers before the intervention commenced. PE teachers from each school were given 8 weeks to complete the delivery of all the lessons and activities during the weekly 30-min health education lessons. Consent was obtained from teachers for delivering the intervention and parents regarding data collection from their children. Students whose parents or guardians did not provide consent for data collection were allowed to attend the lesson but were not included in the data collection.

#### Data collection

**Fig. 3:**
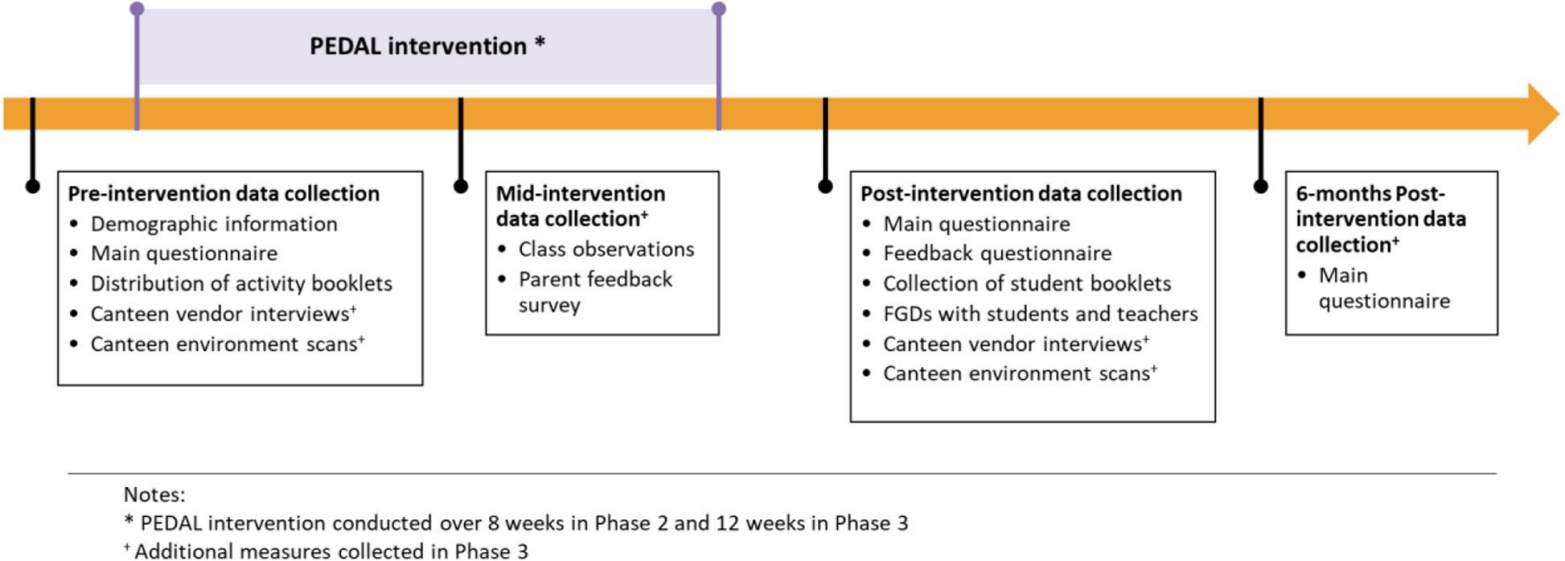
Data collection timeline in Phase 2 and Phase 3

Figure 3 depicts the data collection timeline. Data collection was conducted at two-time points for students—before the intervention commences and after the intervention period. At pre-intervention, demographic information, such as age and gender, and the baseline behaviours and psychosocial factors were collected from students using a questionnaire (“Main questionnaire”). At post-intervention, students completed the post-intervention main questionnaire (consisting of the same questions as in pre-intervention) and a short feedback survey and were invited to participate in an FGD. Teachers also completed a feedback survey after delivering each lesson. All data collected were delinked from their personal information and replaced with a unique code.

##### Main questionnaire

This questionnaire consisted of questions to assess student’s knowledge; ideation factors (attitudes, perceived norms, self-efficacy, social support); and environment related to healthy eating and physical activity. These questions were adapted from validated questionnaires [48, 49]. Behavioural outcomes, such as intentions, habit strength, fruit and vegetable intakes, and physical activity and sedentary time spent, were also captured using the main questionnaire. Details on behavioural outcomes questions are described in the next section.

##### Feedback surveys

Student’s feedback survey consisted of questions on their acceptance (e.g. “I like this style of health education lessons.”) and satisfaction (e.g. “I understood what was taught in the lessons.”, “The lessons have made me more interested to eat healthily and be physically active.”) of the intervention. Teacher’s feedback surveys consisted of a checklist of activities implemented during the lesson, their satisfaction with the instructions provided in the teacher’s guide, their acceptance of facilitating this interactive format of health education lesson, etc.

##### Focus group discussions (FGD)

Two groups of four to six students who participated in the intervention and a group of at least six teachers who delivered the intervention were selected to participate in the FGD from each school. Semi-structured topic guides were used in the FGDs, which included questions to explore healthy eating and physical activity ideation among students and in-depth feedback on the intervention implementation. The topic guide for FGDs with students focused more on exploring ideation, while FGDs with teachers focused more on the implementation.

#### Data analysis

Descriptive statistics were tabulated using frequencies, means, and standard deviations from the quantitative data (e.g. recruitment and participation rates, acceptability and satisfaction rates, online questionnaires) separately for pre-intervention and post-intervention data. Responses from the main questionnaire were examined for ceiling effect. Qualitative data from FGDs were transcribed and analysed using applied thematic analysis, both inductively and deductively [50], to explore the implementation of components A and B and ideation.

The findings and feedback from this phase informed the refinement of the intervention for Phase 3, including extending the intervention period, having more interactive time in class, rephrasing some questions for clarity, and modifying the response options of diet and movement outcomes to capture these behaviours with minimal ceiling effects better. Reported results from Phase 2 will be published separately.

### Phase 3: Pilot testing of the whole PEDAL intervention

#### Objectives

**Fig. 4:**
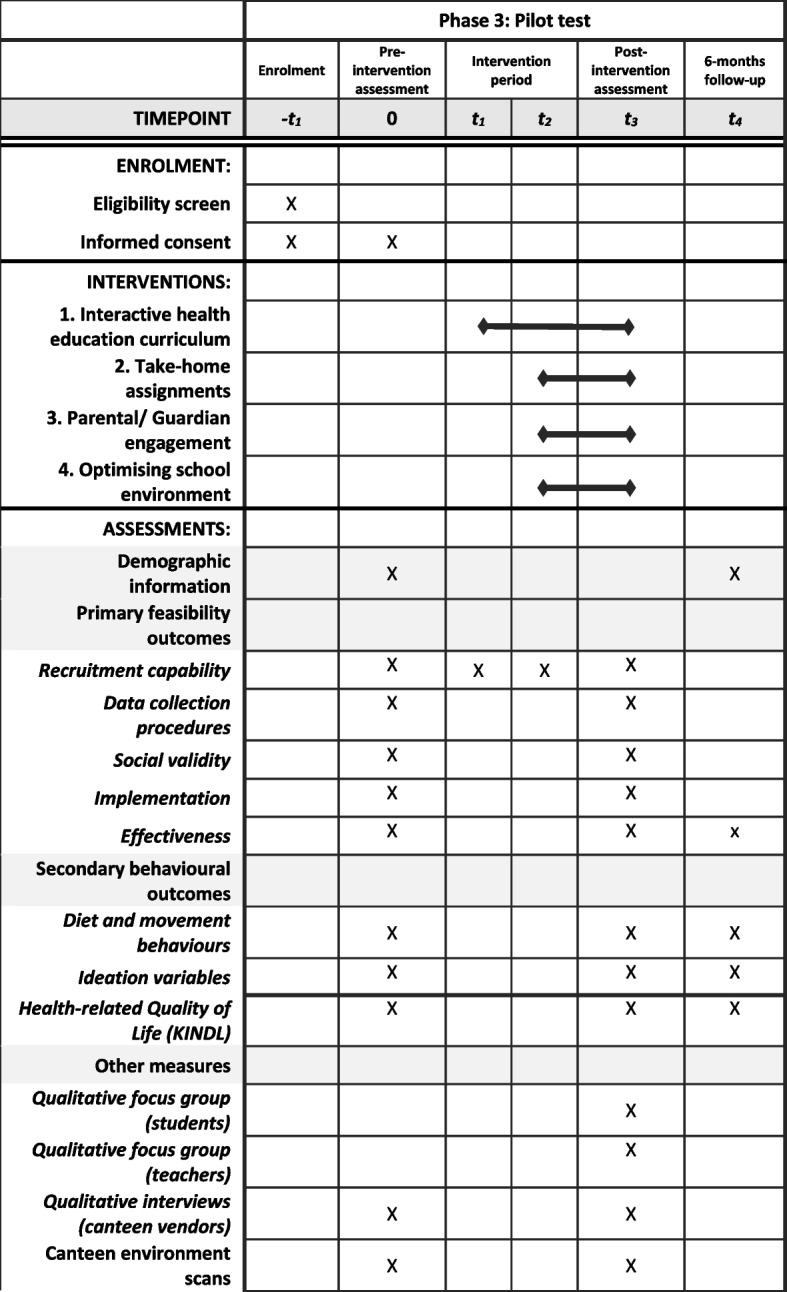
Schedule of enrolment, interventions, and assessments of the pilot study

A pilot study was conducted in Phase 3 to assess the feasibility of the whole PEDAL intervention (components A to D) and explore the potential effectiveness to inform the planning for a future large-scale definitive trial. A concurrent mixed methods quasi-experimental study design was used for this pilot study. Figure 4 illustrates the enrolment, intervention components, and assessment schedule for Phase 3.

#### Key measures

##### Feasibility

Similar to Phase 2, the five feasibility dimensions were assessed with the addition of a sixth dimension, potential effectiveness, which examines if there is preliminary evidence that the intervention has the potential to bring about positive behaviour change. Changes in behavioural outcomes will also be explored to inform the decision-making of the primary outcome and power calculation for a subsequent larger definitive trial. Additionally, FGDs will be conducted with students and teachers to explore any perceived behaviour improvements among the students. With the addition of components C and D, additional measures were also collected from canteen vendors and parents and on the class and canteen environments. Details of the feasibility dimensions and the progression criteria to the next phase (future definitive trial) are described in Table 3.

##### Behavioural outcomes

1. Diet and movement behaviours: Key dietary behaviours to be assessed include fruit and vegetable intakes (in servings per day), while movement behaviours will include time spent on sedentary activities and physical activities (in minutes per day). Students will be asked to report, via a questionnaire, their frequency and usual portion size of their fruit and vegetable intakes in the past week, as well as the time spent on moderate-to-vigorous physical activity (MVPA) and sedentary activities (using screen time as a proxy) in the past week. In addition, students will also complete a 3-day activity record on a web-based application (MEDAL), which was developed by the research team [13] and previously validated against self-reported intakes of school meals [51] and wrist-worn accelerometers [52], to complement the data obtained from the questionnaire.
2. Behavioural intentions: Five items will be used to assess students’ intentions to meet the daily recommended guidelines for fruit intake, vegetable intake, physical activity, and spending less time on sedentary activities in the next three months. A 5-point Likert scale (1, not true for me at all to 5, very true of me) will be used to assess these questions.
3. Habit strength: To assess change in the behavioural habits among students, four items will be used to measure the habit strength in each lifestyle behaviour (i.e. fruit intake, vegetable intake, physical activity, sedentary behaviour). The questions will be adapted from past validated questionnaires [53, 54].
4. Health-related quality of life: In Phase 3, the study will also assess changes in children’s health-related quality of life as previous research has shown that improvements in diet and physical activity levels can contribute to improvements in health-related quality of life in children (HRQoL) (63, 64). As this intervention aims to change children’s lifestyle behaviours, we are interested in examining whether the intervention affects children’s HRQoL. KINDL-Kid (65, 66), which comprised 24 items and six subscales (physical health, general health, family functioning, self-esteem, social function, and school functioning), will be used to measure HRQoL in this study.

#### Setting and participants

The setting, selection of schools, and participants for this pilot study followed the same procedures as Phase 2. However, two other schools were approached to participate in this study. The ideal number of participants for this phase was 385 students, following the same calculation in Phase 2.

Based on the feedback from Phase 2, more time was needed to complete the series of health education lessons in addition to the scheduled physical and health education lessons. Therefore, the schools in this pilot study were given 12 weeks to complete all the lessons and activities. Like Phase 2, consent was obtained from teachers for delivering the intervention and parents regarding data collection from their children. Students whose parents or guardians did not provide consent for data collection were allowed to attend the lesson without any data collected from them.

#### Data collection

The data collection timeline for this phase is illustrated in Fig. 3. Data collection for Phase 3 was similar to Phase 2 with the addition of a mid-intervention, a 6-month post-intervention data collection, and additional measures collected from parents and canteen vendors.

The instruments used for quantitative measurements in Phase 3 were largely similar to those used in Phase 2, with some refinements made to the phrasing and response options in the main questionnaire. A feedback survey for parents was also developed to assess parent’s feedback on the lesson videos and home activities. An observation checklist of the canteen environment was also developed to examine the availability of healthier choices, the canteen vendor’s compliance with the Healthy Meals in School Programme guidelines [44], the interaction between canteen vendors and students, etc.

The selection of participants for the focus groups was the same as in Phase 2. Different semi-structured topic guides were developed for Phase 3 to assess the extent of students’ and teachers’ awareness and knowledge retained from the PEDAL lessons, collect feedback on the whole PEDAL intervention, and explore perspectives on behaviour change and habit formation in students. For the short interviews with canteen vendors, guiding questions were developed to explore their perspective of the school food environment and perceived changes among students after the intervention. All data collected were delinked from their personal information and replaced with a unique code.

#### Data analysis

Descriptive statistics from the quantitative data for the recruitment capability, data collection procedures, and social validity dimensions will also be presented separately for pre-intervention and post-intervention data in this phase. Pre-post intervention comparisons of the behavioural outcomes will be assessed using appropriate statistical analyses (e.g. paired *t*-test for normally distributed variables and Wilcoxon signed rank test for variables with non-parametric distributions). Pre- and post-intervention ideation scores for healthy eating and physical activity, derived from the composite of the ideation variable scores, will be compared. Regression analyses will be conducted to examine the associations between ideation scores and behavioural outcomes.

Similar to Phase 2, qualitative data from FGDs and interviews will be transcribed and analysed using applied thematic analysis [50], to answer the research questions for the social validity, practicality, implementation, and (perceived) potential effectiveness dimensions. When possible, both quantitative and qualitative data will be integrated and reported for the feasibility evaluation.

## Dissemination strategy

Findings from this study will be disseminated to key stakeholders, such as MOE, HPB, the schools, and the parents of children who participated in the study. Findings from this study will be disseminated through at least two publications and will form part of the theses for a PhD student and a Master’s student. The improved version of intervention materials will also be shared with MOE for use as future resources for health education lessons.

## Discussion

Multiple health promotion programmes have been conducted in local public schools to improve children’s diet and movement behaviours; however, these interventions tend to be of a single component [14, 21]. With the growing body of literature demonstrating the effectiveness of multicomponent interventions, there is a need for multicomponent interventions to support healthy behaviour change among children in Singapore. However, information on the feasibility and acceptability of multicomponent interventions in Singapore is lacking. To our knowledge, this is the first study detailing the development and testing of a theory-driven multicomponent and school-based educational intervention among primary school children that targets both dietary and movement behaviours of children on the individual, interpersonal and environmental levels.

This study seeks to identify probable “active ingredients” of the intervention and understand the factors leading to a successful implementation of a multicomponent school-based intervention through the pilot testing of the PEDAL intervention. However, it should be noted that only two schools were recruited for the feasibility and pilot testing phase of the study which means that the findings may not be generalisable to all schools in Singapore or all co-educational public schools. As funding and resource constraints limited our ability to recruit more schools, only two schools underwent the intervention, and a pretest-posttest design was used. A limitation of such design is that it limits our certainty of how the outcomes might change in the absence of the intervention. Therefore, findings on the potential effectiveness should be interpreted with caution, and a larger-scale definitive trial (e.g. a randomised controlled trial (RCT)) should be conducted to assess the effectiveness of this intervention.

## Study status

The study is prospectively registered with the ISRCTN registry (ISRCTN16114046). The development of PEDAL commenced in April 2021, and the prototype was developed in December 2021 before feasibility was examined (Phase 2). Phase 2 was completed in November 2022, and the manuscript is currently being prepared for submission. The intervention materials were refined in December 2022 for the pilot test (Phase 3). The pilot intervention was completed in November 2023, and data analysis is underway.

### Supplementary Information

**Additional file 1.** SPIRIT 2013 Checklist: Recommended items to address in a clinical trial protocol and related documents*.

**Additional file 2.** Snapshots of developed videos and slides for health education lessons.

**Additional file 3.** Snapshots of the home activity tasks.

## Data Availability

The datasets generated during and/or analysed during the current study will be available upon request from Dr Mary Chong after publication.
