# Supplementary figures and images for: Promoting hEalthy Diet and Active Lifestyle (PEDAL): a protocol for the development and feasibility study of a multicomponent intervention among primary school children in Singapore
Source: Pilot Feasibility Stud. 2024 Mar 23;10:52. doi: 10.1186/s40814-024-01479-3 (PMC10960416; doi:10.1186/s40814-024-01479-3)

Additional file 2. Snapshots of developed videos and slides for health education lessons


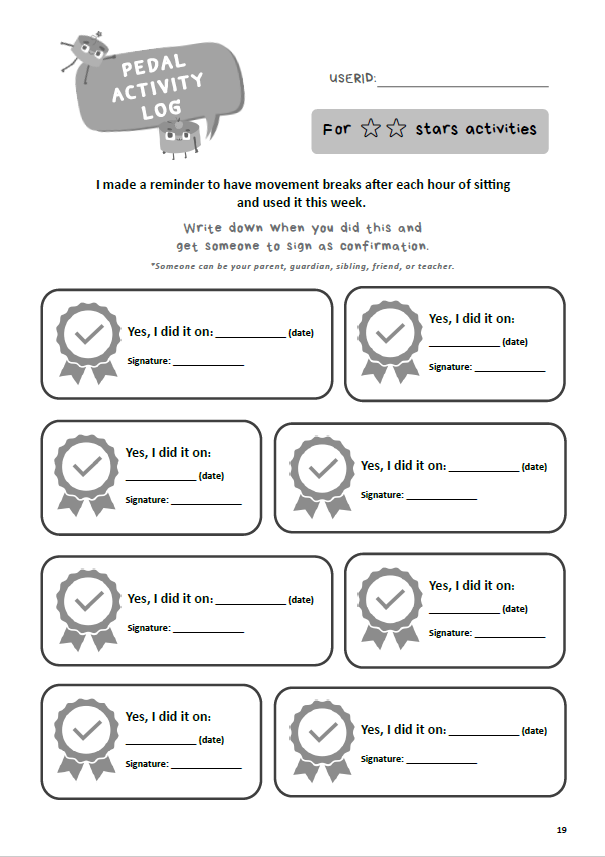

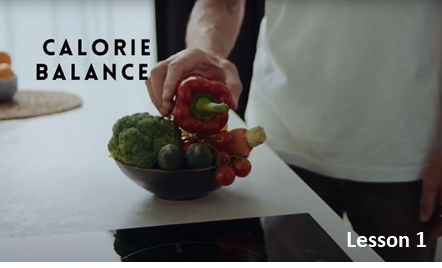

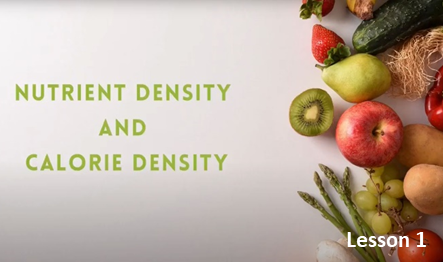

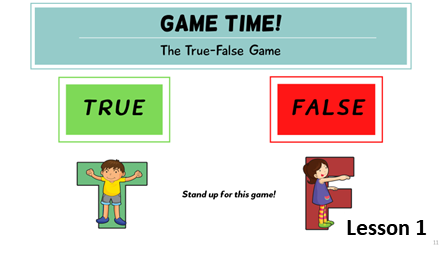


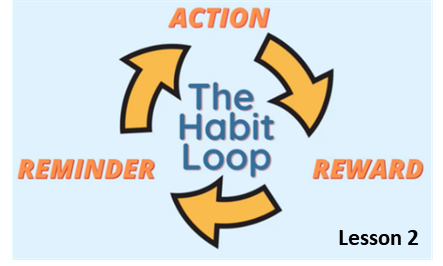

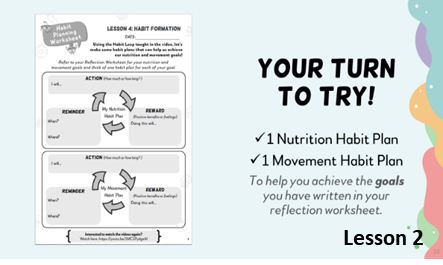

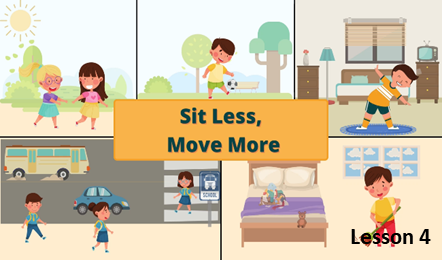

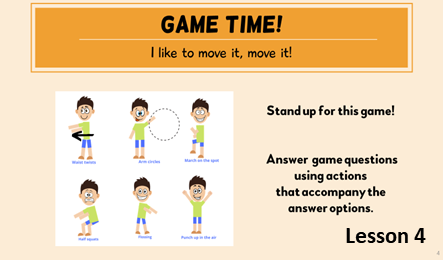

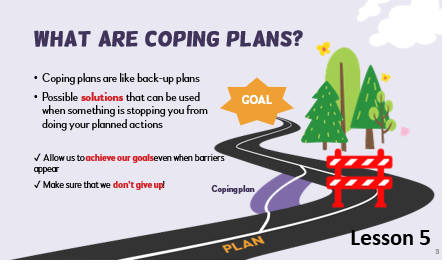

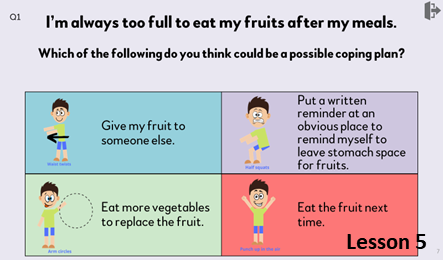

Supplement: Supplementary file 2 — Additional file 2. Snapshots of developed videos and slides for health education lessons. [file 40814_2024_1479_MOESM2_ESM.docx]

Additional file 3. Snapshots of home activity tasks


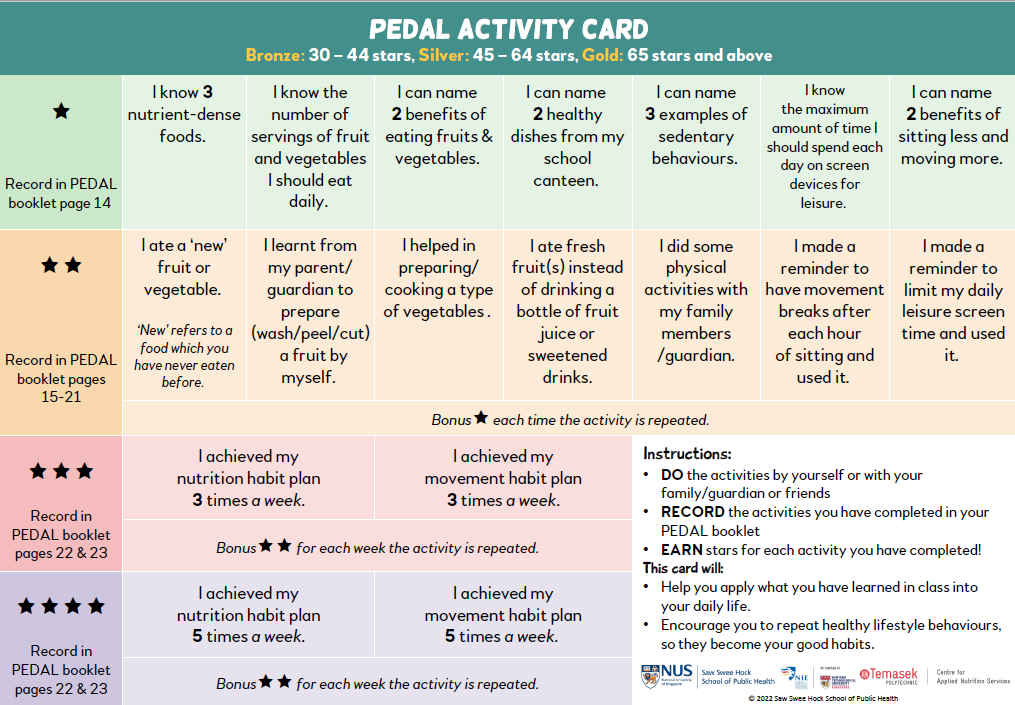

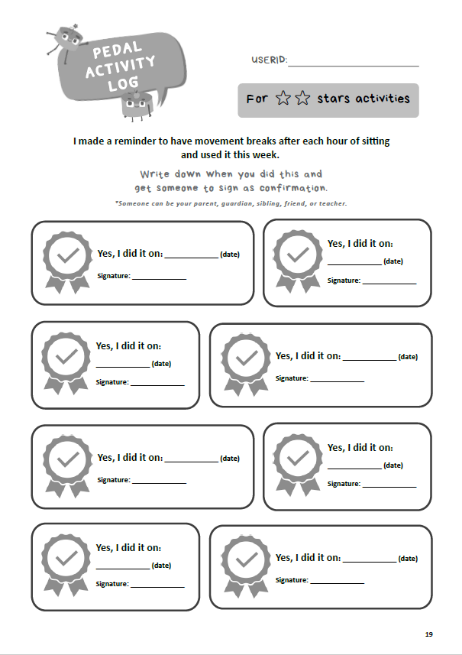

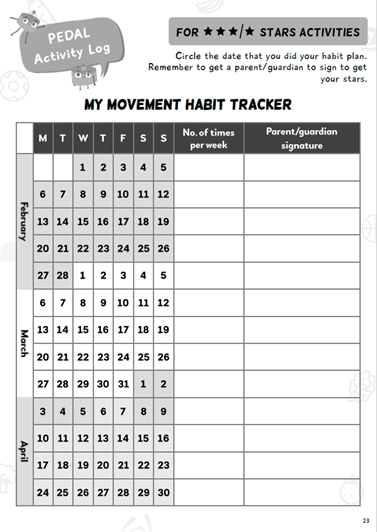

Supplement: Supplementary file 3 — Additional file 3. Snapshots of the home activity tasks. [file 40814_2024_1479_MOESM3_ESM.docx]
